# Prognostic value of metastatic lymph node ratio and its effect on disease-free survival in colon cancer

**DOI:** 10.3389/fonc.2025.1624798

**Published:** 2025-08-27

**Authors:** Orhan Aslan, Ramazan Topcu, İsmail Sezikli, Mahmut A. Yüksek, Aşkın K. Perçem, Furkan Uğur

**Affiliations:** ^1^ Department of General Surgery, Faculty of Medicine, Hitit University, Çorum, Türkiye; ^2^ Department of Gastroenterology Surgery, Hitit University Erol Olçok Training and Research Hospital, Çorum, Türkiye

**Keywords:** colon cancer, metastatic lymph node ratio, prognosis, survival, disease recurrence, adjuvant chemotherapy

## Abstract

**Introduction:**

The metastatic lymph node ratio (MLNR) has been proposed as a meaningful prognostic indicator in colon cancer (CC). This study aimed to assess the prognostic relevance of MLNR by investigating its association with disease-free survival (DFS), overall survival (OS), and recurrence, and to compare its predictive value with traditional parameters, including the TNM classification and total lymph node count (TNLC).

**Materials and methods:**

This retrospective, single-center study included patients who underwent surgical resection for colon cancer. Survival outcomes were analyzed using Kaplan-Meier survival curves and multivariate logistic regression. MLNR was evaluated in relation to demographic and clinical factors, including age, tumor location, surgical type, and the administration of adjuvant chemotherapy. The optimal MLNR cut-off value for predicting recurrence was determined via receiver operating characteristic (ROC) curve analysis.

**Results:**

A total of 122 patients were analyzed. MLNR >0.125 was significantly associated with increased recurrence risk (adjusted HR: 7.0, p<0.001) and reduced DFS. Patients with an MLNR ≤0.125 demonstrated significantly longer DFS (p<0.001). MLNR emerged as an independent prognostic factor, offering potential prognostic benefit compared to TNLC in predicting both DFS and OS. Additionally, adjuvant chemotherapy was independently associated with a lower recurrence risk (Exp(B):0.234, p=0.038). Emergency surgery was found to be significantly correlated with poorer survival outcomes (p=0.023).

**Conclusion:**

MLNR contributes additional prognostic information to the TNM staging system and may support more individualized risk stratification and decision-making regarding adjuvant therapy in colon cancer. Further large-scale prospective studies are warranted to validate these findings and to establish a clinically applicable MLNR threshold.

## 1 Introduction

Colorectal cancer (CRC) imposes a significant burden on healthcare systems worldwide, with millions of new cases diagnosed annually. According to GLOBOCAN 2020 data, CRC ranks as the third most common cancer globally, with an incidence of approximately 1.9 million cases and accounting for 9.4% of all cancer-related deaths (1, 2). The increasing incidence of CRC has been attributed to an aging population and the widespread adoption of a Western lifestyle. Lymph node dissection in the surgical treatment of CRC is not only a measure of surgical adequacy but also a critical step for accurate staging and determining the need for adjuvant chemotherapy. However, studies indicate that in approximately 30-50% of resections, this standard is not met, which is associated with lower survival rates. Additionally, variability in the number of lymph nodes retrieved during surgery and pathological examination may limit the precision of staging. Accurate staging is crucial for prognostication and treatment planning in colon cancer. The TNM classification system, which stands for Tumor (T), regional lymph Nodes (N), and distant Metastasis (M), is widely used to categorize the extent of disease and to guide therapeutic decisions (3, 4).

In some patients, recurrence after curative resection has been linked to residual disease caused by inadequate lymphadenectomy. Although the National Comprehensive Cancer Network (NCCN) guidelines recommend the pathological evaluation of at least 12 lymph nodes, the number of lymph nodes evaluated in clinical studies varies widely, ranging from 7 to 40 (5, 6). This wide range poses challenges in determining the optimal number of lymph nodes to be examined. The pathological examination of at least 12 lymph nodes may allow the reclassification of cases initially staged as stage 1–2 to stage 3 (3, 7). Using the ratio of metastatic lymph nodes to the total number of lymph nodes (MLNR) examined, independent of the total lymph node count, offers a potential solution to this challenge (8). The relationship between the total number of lymph nodes removed and disease-free survival remains controversial (9, 10). While some authors confirm this association, others emphasize that survival outcomes are influenced not only by accurate staging but also by the extent of the radical surgical approach (11, 12). These ongoing debates highlight the importance of investigating the MLNR.

The MLNR defined as the number of metastatic lymph nodes divided by the total number of lymph nodes retrieved, has been proposed as a sensitive prognostic metric that may offer improved risk stratification compared to conventional nodal count alone. As previously demonstrated by Chen et al. and Cozzani et al., MLNR is considered a more sensitive prognostic tool compared to the traditional TNM classification system (13, 14). Therefore, incorporating MLNR into clinical practice for colon cancer could provide a complementary tool to existing staging systems. Yet, there remains no universally accepted MLNR cut-off value. Thresholds ranging from 0.05 to 0.3 have been explored, often based on institutional data or retrospective analyses. The absence of standardization limits the widespread clinical adoption of MLNR.

Therapeutic innovations in CRC treatment have shown significant progress in recent years across both surgical and medical domains. Minimally invasive surgical techniques and optimized lymph node dissection strategies have improved oncologic outcomes, while targeted therapies and immunotherapeutic agents have expanded systemic treatment options. Notably, the integration of prognostic indicators such as the MLNR into these evolving treatment approaches has attracted increasing attention (15, 16).

This study aims to investigate the prognostic value of MLNR in patients with surgically treated colon cancer and to assess whether it provides additional prognostic information beyond traditional TNM staging. We also explore the relationship between MLNR and recurrence risk, as well as the potential role of MLNR in guiding adjuvant chemotherapy decisions. By identifying an optimal MLNR cut-off value, our goal is to support more individualized postoperative management strategies in colon cancer.

## 2 Materials and methods

### 2.1 Study administration

This retrospective, single-center study was conducted at the General Surgery Department of Hitit University Erol Olçok Training and Research Hospital. Data from all patients aged 18 years or older who underwent surgery for colon cancer between January 1, 2016, and January 1, 2022, were reviewed using the hospital’s information system. Given the retrospective nature of the study and anonymized data collection, the requirement for informed consent was waived by the Clinical Research Ethics Committee (Protocol No: 2023-60, 24.05.2023), in accordance with institutional and international ethical standards. Exclusion criteria included patients with distant organ metastases at the time of diagnosis, those with known oncologic diseases other than newly diagnosed colon cancer, those who underwent palliative surgery, patients with incomplete data, and those under 18 years of age. Additionally, cases with rectal tumors were excluded due to differences in treatment protocols. However, tumors located in the rectosigmoid junction or distal sigmoid colon, which were managed using colon cancer protocols, were included in the analysis. These tumors may have required anterior or low anterior resection depending on their proximity to the rectum, which explains the proportion of such surgical procedures in the cohort. Although patients with stage 0 colon cancer do not typically have lymph node involvement, they were retained in the overall cohort for completeness. However, they were excluded from subgroup analyses where MLNR or nodal metastasis was the primary variable of interest. This approach preserved statistical power while minimizing bias. Ethical approval for this study was obtained from the Clinical Research Ethics Committee of Hitit University Faculty of Medicine (Protocol No: 2023-60) and the study protocol was conducted in accordance with the 1964 Helsinki Declaration.

A total of 122 patients who met the inclusion criteria were enrolled in the study. All patients included in the study were of Turkish ethnicity, as the data were collected from a single tertiary healthcare institution in Türkiye. This demographic context should be considered when interpreting the generalizability of the results. Data collected included demographic characteristics, tumor location, total and metastatic lymph node counts retrieved during surgery, disease stage, recurrence and mortality status during follow-up, length of hospital stay, chemotherapy treatments, postoperative complications, and follow-up durations.

### 2.2 Definitions

MLNR was calculated as the number of metastatic lymph nodes divided by the total number of lymph nodes retrieved during surgery.

Disease-free survival (DFS) was defined as the time from surgery to the date of documented recurrence or last follow-up without recurrence. Overall survival (OS) was defined as the time from surgery to death from any cause or last follow-up.

### 2.3 Data analysis

All statistical analyses were performed using IBM SPSS Statistics for Windows, version 26 (IBM Corp., Armonk, NY, USA). Descriptive statistics were used to summarize categorical variables as frequencies and percentages, while numerical variables were expressed as mean ± standard deviation or median, depending on the distribution. The normality of data distribution was assessed using the Shapiro-Wilk test. Relationships between variables were analyzed using Pearson or Spearman correlation coefficients based on data distribution. Comparisons of numerical variables between study groups were conducted using the Mann-Whitney U test for all variables except age, which was analyzed using the Student’s t-test based on a Gaussian distribution. Categorical variables were compared using the Chi-square test.

Interactions between variables were examined through binomial logistic regression analysis to evaluate the independent predictive capacity of the MLNR for recurrence. Confounding variables were identified based on both clinical relevance and statistical significance in univariate analysis (p<0.10). These variables were entered into a multivariate logistic regression model to evaluate the independent effect of MLNR on recurrence, adjusting for potential confounders such as age, comorbidities, emergency surgery, operation type, and adjuvant therapy. The model demonstrated good fit (Nagelkerke R²=0.278, classification accuracy: 84.4%).

In calculating the MLNR, all retrieved lymph nodes from the surgical specimen were included in the denominator, regardless of their anatomical location. However, we acknowledge that this approach may introduce bias, especially in right hemicolectomy specimens, where lymph nodes from the midcolic area may be included alongside truly regional nodes such as the ileocolic group. Although such inclusion was done for consistency and feasibility in this retrospective study, we recognize the potential influence of anatomical variability on MLNR values.

Receiver Operating Characteristic (ROC) curve analysis was used to evaluate the discriminative ability of MLNR in predicting recurrence. Several cut-off values in the range of 0.10 to 0.15 were tested during ROC analysis. Among these, 0.125 was selected as the optimal threshold based on the highest Youden index value, providing the best trade-off between sensitivity (61.9%) and specificity (83.2%) for predicting recurrence. Diagnostic performance metrics, including sensitivity, specificity, positive predictive value (PPV), negative predictive value (NPV), accuracy, and odds ratios were calculated for this cut-off.

Kaplan-Meier survival analysis was performed to evaluate disease-free survival, and statistical significance between groups was assessed using the Log-Rank test. A p-value of <0.05 was considered statistically significant.

## 3 Results

### 3.1 Baseline characteristics

The study included a total of 122 patients, of whom 44 (36.07%) were female and 78 (63.93%) were male. The median age was 68.46 ± 10.61 years. Among the patients, 73 (59.84%) underwent elective surgeries. The mean DFS duration was 36 months, ranging from 6 to 72 months. During the 60-month follow-up period, 25 patients (20.49%) died. Detailed demographic and clinical variables are summarized in **Table 1**.

**Table 1:** Demographic and follow up features.

	All Patients (n=122)	Alive (n=97)	Deceased (n=25)	p-value
Gender, N (%)				
Female	44 (36.07%)	38 (39.18%)	6 (24%)	0.159^a^
Male	78 (63.93%)	59 (60.82%)	19 (76%)	
Age (mean ± SD)	68.46 ± 10.61	66.48 ± 9.56	76.12 ± 11.2	<0.001^b^
Comorbidity, N (%)	78 (63.93%)	59 (60.82%)	19 (76%)	0.159^a^
Follow-Up Characteristics				
Hospitalization Duration, Days	13.5 (4-59)	14 (4-59)	13 (7-36)	0.822^c^
Follow-Up Duration, (months)	36 (12-72)	48 (12-72)	36 (12-60)	0,002^c^
Disease-Free Survival Duration (months)	36 (6-72)	48 (6-72)	18 (6-48)	<0.001^c^
Overall Survival Duration (months)	36 (12–72)	48 (12–72)	36 (12–60)	0,002^c^

^a^Chi-square or Fisher’s exact test, ^b^Student’s t-test, ^c^Mann–Whitney U test. Values are presented as median (range), mean ± SD, or N (%), as appropriate.

### 3.2 Mortality analysis

Patients were categorized into two groups based on survival status: alive (n=97) and deceased (n=25). While no statistically significant difference was observed in gender distribution (p=0.159), age showed a significant association with mortality. The median age of surviving patients was 66.48 ± 9.56 years, compared to 76.12 ± 11.2 years for those who had died (p<0.001; **Table 1**).

Emergency surgery and tumor localization were significantly associated with mortality in univariate analyses (p=0.023 and p=0.009, respectively). However, operation type, histopathology, disease stage, and length of hospital stay did not show significant associations with mortality (p=0.108, p=0.952, p=0.836, and p=0.822, respectively; **Table 2**). Treatment and follow-up factors were also evaluated. Local recurrence, distant metastasis, and overall recurrence were significantly associated with mortality (p<0.001, p=0.008, and p<0.001, respectively). However, adjuvant therapy, the number of malignant lymph nodes, and total lymph node count were not significant predictors (p=0.697, p=0.082, and p=0.802, respectively; **Table 2**). Complications during treatment were not significantly associated with mortality (p=0.507) (**Table 2**). Contrary to our initial hypothesis, MLNR was not significantly different between survivors and deceased patients.

**Table 2:** Clinical and surgical features.

	All Patients (n=122)	Alive (n=97)	Deceased (n=25)	p-value
Timing of Surgery, N (%)				
Emergent	49 (40.16%)	34 (35.05%)	15 (60%)	0.023^a^
Elective	73 (59.84%)	63 (64.95%)	10 (40%)	
No Complication, N (%)	107 (87.7%)	86 (88.66%)	21 (84%)	0.507 ^a^
No Adjuvant Therapy, N (%)	53 (43.44%)	43 (44.33%)	10 (40%)	0.697 ^a^
Tumor Localization, N (%)				
Rectosigmoid	28 (22.95%)	17 (17.53%)	11 (44%)	0.009 ^a^
Sigmoid	29 (23.77%)	22 (22.68%)	7 (28%)	
Descending	17 (13.93%)	17 (17.53%)	0 (0%)	
Transverse	11 (9.02%)	11 (11.34%)	0 (0%)	
Ascending	37 (30.33%)	30 (30.93%)	7 (28%)	
Operation Type, N (%)				
Low anterior resection	30 (24.59%)	20 (20.62%)	10 (40%)	0.108 ^a^
Anterior resection	24 (19.67%)	18 (18.56%)	6 (24%)	
center hemicolectomy	24 (19.67%)	22 (22.68%)	2 (8%)	
Right hemicolectomy	44 (36.07%)	37 (38.14%)	7 (28%)	
Histopathology, N (%)				
*In situ* adenocarcinoma	2 (1.64%)	2 (2.06%)	0 (0%)	0.952 ^a^
Well-differentiated	31 (25.41%)	24 (24.74%)	7 (28%)	
Moderately-differentiated	72 (59.02%)	57 (58.76%)	15 (60%)	
Poorly-differentiated	6 (4.92%)	5 (5.15%)	1 (4%)	
Mucinous adenocarcinoma	11 (9.02%)	9 (9.28%)	2 (8%)	
Stage, N (%)				
0*	2 (1.64%)	2 (2.06%)	0 (0%)	0.836 ^a^
I	8 (6.56%)	7 (7.22%)	1 (4%)	
II	56 (45.90%)	45 (46.39%)	11 (44%)	
III	56 (45.90%)	43 (44.33%)	13 (52%)	
Recurrence				
Local recurrence, N (%)	14 (11.48%)	4 (4.12%)	10 (40%)	<0.001 ^a^
Metastatic, N (%)	12 (9.84%)	6 (6.19%)	6 (24%)	0.008 ^a^
Overall, N (%)	21 (17.21%)	8 (8.25%)	13 (52%)	<0.001 ^a^
Malignant LN, N (range)	0 (0-12)	0 (0-12)	1 (0-12)	0.082 ^b^
Total LN, N (range)	15 (0-61)	16 (1-61)	15 (0-48)	0.802 ^b^
MLNR, N (range)	0 (0-1)	0 (0-0.85)	0.02 (0-1)	0.138 ^b^

LN, lymph node; MLNR, metastatic lymph node ratio.

^a^ Chi-square or Fisher’s exact test, ^b^ Mann-Whitney U test. Values are presented as median (range), mean ± SD, or N (%), as appropriate.

*Two stage 0 patients were included in overall demographic summaries but excluded from MLNR-based subgroup analyses and regression models due to absence of nodal metastasis risk.

### 3.3 Recurrence analysis

Patients were then stratified into recurrence (n=21) and non-recurrence (n=101) groups. Gender distribution did not differ significantly between the groups (p=0.199). Age was significantly associated with mortality, whereas no statistically significant association was found between age and recurrence (p=0.935). The presence of comorbidities (p=0.774), emergency surgery (p=0.444), tumor localization (p=0.075) and type of surgical intervention (p=0.170) showed no significant associations with recurrence.

Disease staging was significantly associated with recurrence (p=0.034). Although adjuvant therapy was more frequently administered in the recurrence group (p=0.015), this likely reflects clinical decisions informed by higher initial risk profiles or unfavorable prognostic factors, rather than implying a direct causal relationship with recurrence. Complications during treatment were not significantly associated with recurrence (p=0.135). Patients without recurrence had significantly longer follow-up durations compared to those with recurrence (p=0.011), likely due to higher mortality rates in the recurrence group (11.88% vs. 61.9%, p<0.001). The presence of malignant lymph nodes was significantly associated with recurrence (p<0.001). Although the total lymph node count was not a significant predictor (p=0.273), MLNR emerged as a highly significant factor in univariate analysis (p<0.001). A full comparison of clinical and pathological variables between recurrence groups is presented in **Table 3**.

**Table 3:** Comparison of clinical and surgical features by recurrence status.

	No Recurrence (n=101)	Recurrence (n=21)	p-value
Gender: Male	62 (61.39%)	16 (76.19%)	0.199 ^a^
Gender: Female	39 (38.61%)	5 (23.81%)	
Age (mean ± SD)	68.5 ± 10.67	68.29 ± 10.56	0.935 ^b^
Presented Comorbidity, N (%)	64 (63.37%)	14 (66.67%)	
Timing of Surgery, N (%)			
Emergent	39 (38.61%)	10 (47.62%)	0.444 ^a^
Elective	62 (61.39%)	11 (52.38%)	
Localization, N (%)			
Rectosigmoid	19 (18.81%)	9 (42.86%)	0.075 ^a^
Sigmoid	25 (24.75%)	4 (19.05%)	
Descending	16 (15.84%)	1 (4.76%)	
Transverse	11 (10.89%)	0 (0%)	
Ascending	30 (29.7%)	7 (33.33%)	
Operation Type, N (%)			
Low anterior resection	22 (21.78%)	8 (38.1%)	0.170^a^
Anterior resection	19 (18.81%)	5 (23.81%)	
center hemicolectomy	23 (22.77%)	1 (4.76%)	
Right hemicolectomy	37 (36.63%)	7 (33.33%)	
Histopathology, N (%)			
*In situ* adenocarcinoma	2 (1.98%)	0 (0%)	0.900^a^
Well-differentiated	25 (24.75%)	6 (28.57%)	
Moderately-differentiated	59 (58.42%)	13 (61.9%)	
Poorly-differentiated	5 (4.95%)	1 (4.76%)	
Mucinous adenocarcinoma	10 (9.9%)	1 (4.76%)	
Stage, N (%)			
0*	2 (1.98%)	0 (0%)	0.034 ^a^
I	8 (7.92%)	0 (0%)	
II	51 (50.5%)	5 (23.81%)	
III	40 (39.6%)	16 (76.19%)	
Local Recurrence, N (%)	0 (0%)	14 (66.67%)	<0.001^a^
Distant Organ Metastasis, N (%)	0 (0%)	12 (57.14%)	<0.001^a^
Malignant LN, N (range)	0 (0-12)	3 (0-12)	<0.001^c^
Total LN, N (range)	15 (0-61)	17 (5-48)	0.273 ^c^
MLNR, N (range)	0 (0-0.85)	0.16 (0-1)	<0.001^c^
No Complication, N (%)	91 (90.1%)	16 (76.19%)	0.135 ^a^
Adjuvant Therapy: No	49 (48.51%)	4 (19.05%)	0.015 ^a^
Adjuvant Therapy: Yes	52 (51.49%)	17 (80.95%)	
Mortality: Deceased	12 (11.88%)	13 (61.9%)	<0.001^a^
Disease-Free Survival Duration (months)	48 (12-72)	12 (6-36)	<0.001^a^
Overall Survival Duration (months)	48 (12-72)	36 (12-60)	0.011^a^

LN, lymph node; MLNR, metastatic lymph node ratio; mo, months.

^a^ Chi-square or Fisher’s exact test, ^b^ Student’s t-test, ^c^ Mann-Whitney U test. Values are presented as median (range), mean ± SD, or N (%), as appropriate.

*Two stage 0 patients were included in overall demographic summaries but excluded from MLNR-based subgroup analyses and regression models due to absence of nodal metastasis risk.

### 3.4 Multivariate analysis

To determine whether MLNR independently predicted recurrence, multivariate logistic regression analysis was performed, incorporating variables identified as clinically relevant or statistically significant in univariate analysis. These included age, gender, comorbidities, surgical urgency (emergency vs. elective), type of surgery, postoperative complications, use of adjuvant chemotherapy, and MLNR (categorized by the cut-off value of 0.125). As shown in **Table 4**, MLNR ≥ 0.125 emerged as an independent predictor of disease recurrence (odds ratio [OR]: 13.07; 95% confidence interval [CI]: 1.03-166.57; p=0.048). Conversely, receipt of adjuvant therapy was associated with a significant reduction in recurrence risk (OR: 0.234; 95% CI: 0.059-0.923; p=0.038). Other factors such as age, gender, and surgical urgency did not retain statistical significance in the final model.

**Table 4:** Univariate and multivariate analysis of overall recurrence.

	Univariate analysis p-value	Multivariate OR [95% CI]	Multivariate p-value
Gender	0.199	0.487 [0.144-1.645]	0.247
Age	0.935	0.989 [0.932-1.049]	0.716
Comorbidity	0.774	0.504 [0.145-1.749]	0.281
Emergent Surgery	0.444	1.375 [0.435-4.343]	0.588
Operation Type	0.170	1.454 [0.347-6.098]	0.608
Complication	0.011	0.741 [0.170-3.230]	0.690
MLNR	<0.001	13.072 [1.026-166.565]	0.048
Adjuvant Therapy	0.015	0.234 [0.059-0.923]	0.038

MLNR, metastatic lymph node ratio.

### 3.5 Optimal MLNR cut-off and survival analysis

**Figure 1:**
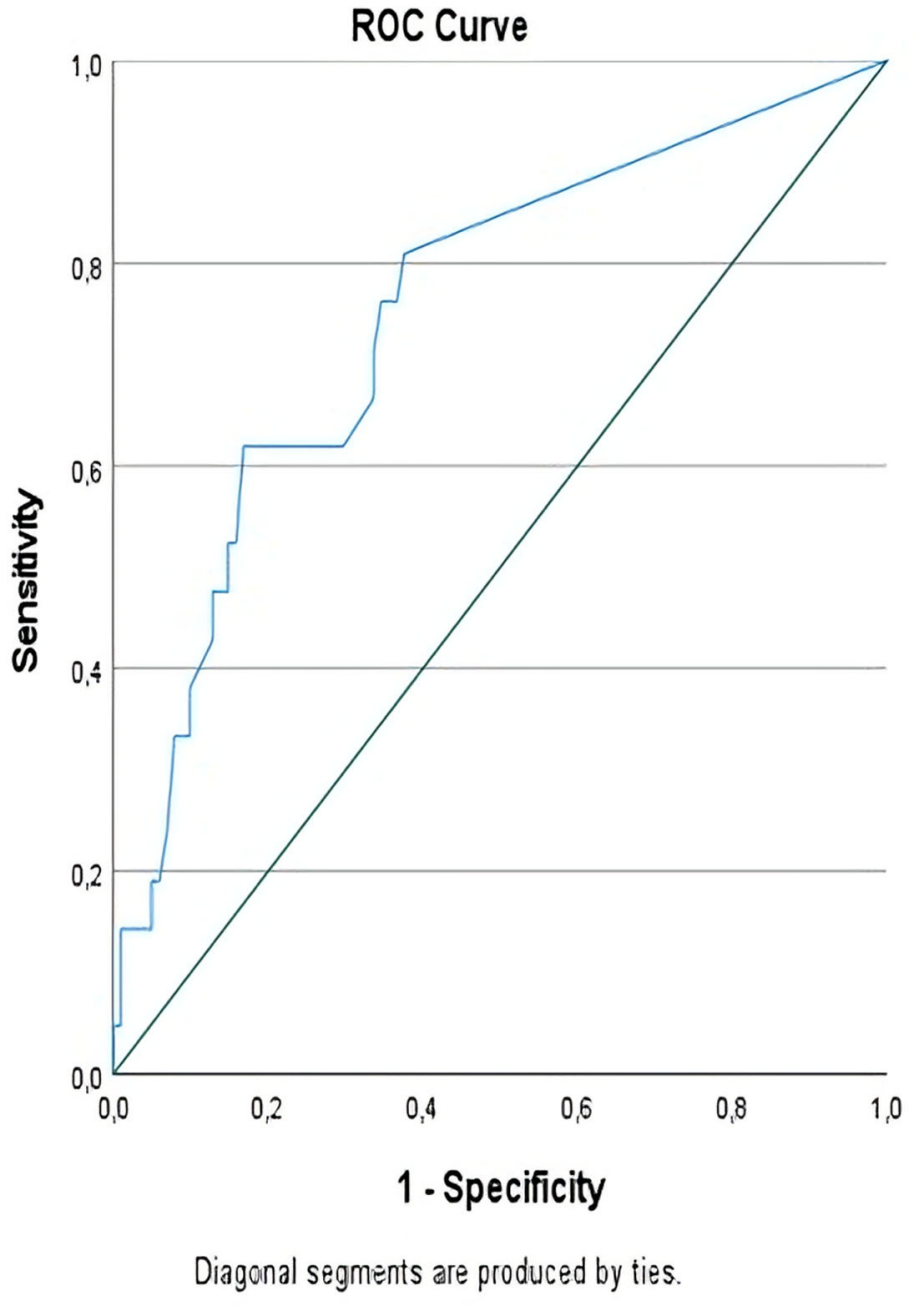
ROC Analysis of MLNR cut-off value for predicting recurrence.

The discriminative ability of MLNR in predicting recurrence was further evaluated using ROC curve analysis. The area under the ROC curve (AUC) was 0.752 (95% CI: 0.634-0.869; p < 0.001), indicating good prognostic performance. The optimal MLNR threshold for recurrence prediction was identified as 0.125 using the Youden Index. At this cut-off, MLNR had a sensitivity of 61.9% and a specificity of 83.2%. The positive predictive value (PPV) and negative predictive value (NPV) were 43.3% and 91.3%, respectively, yielding an overall diagnostic accuracy of 79.5%. The odds of recurrence in patients with MLNR ≥ 0.125 were significantly higher compared to those below this threshold (OR: 8.03; 95% CI: 2.89-22.34; p < 0.001). These diagnostic performance metrics are presented in **Table 5**, and the ROC curve is illustrated in **Figure 1**.

**Table 5:** Diagnostic performance of MLNR cut-Off (0.125) for recurrence prediction.

Metric	Value
Sensitivity	61.9%
Specificity	83.2%
Positive Predictive Value (PPV)	43.3%
Negative Predictive Value (NPV)	91.3%
Accuracy	79.5%
AUC (95% CI)	0.752 (0.060), 95% CI: 0.634–0.869
p-value (ROC)	<0.001
Odds Ratio	8.029 (95% CI: 2.885–22.343), p < 0.001

MLNR, metastatic lymph node ratio.

### 3.6 Kaplan-Meier survival analysis

**Figure 2:**
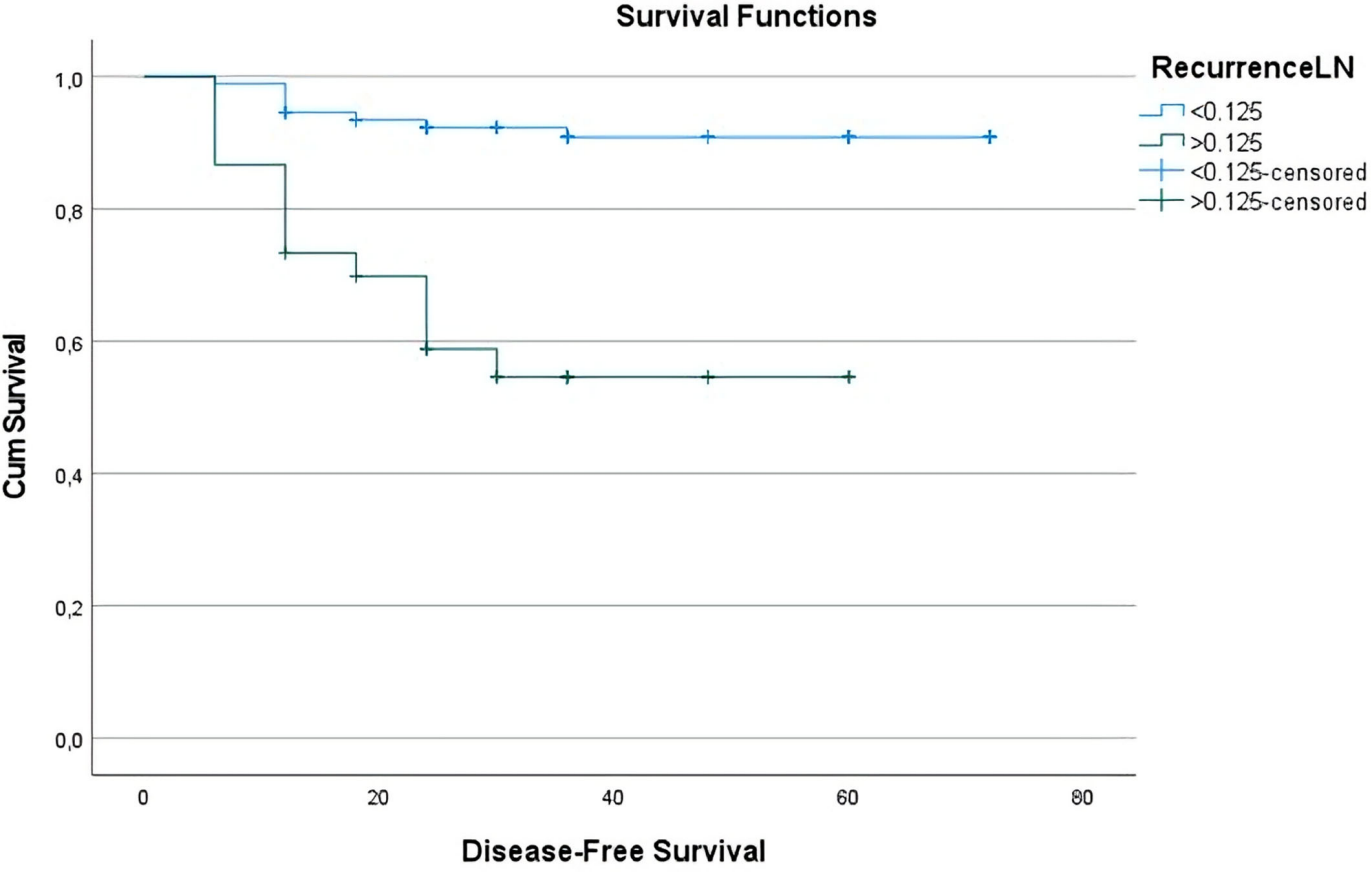
Disease-free survival curve based on MLNR ratios. The overall mean estimated DFS duration for the entire cohort was 61.93 months.

DFS was further analyzed using Kaplan-Meier survival curves stratified by MLNR groups. Patients with MLNR < 0.125 demonstrated significantly longer DFS compared to those with MLNR≥0.125 (log-rank p < 0.001). The mean DFS duration was 42.13 ± 17.9 months in the MLNR <0.125 group, while it was 27.00 ± 15.66 months in the MLNR≥0.125 group. The estimated mean DFS for the low MLNR group was 66.99 months (95% CI: 63.66-70.32), in contrast to 39.70 months (95% CI: 31.32-48.08) for the high MLNR group. The overall cohort had an estimated mean DFS of 61.93 months (95% CI: 57.99-65.87), as summarized in **Table 6** and visualized in **Figure 2**. These findings underscore the prognostic value of MLNR in stratifying patients according to recurrence risk and disease-free survival. Although MLNR was significantly associated with DFS in Kaplan-Meier analysis, we did not perform Cox proportional hazards modeling to compute hazard ratios, as our primary survival outcome analysis was categorical (recurrence vs. no recurrence) and based on logistic regression.

**Table 6:** Estimated disease-free survival by MLNR groups.

MLNR Group	Estimated Mean DFS (95% CI)	Standard Error	Statistical Significance (log-rank)
< 0.125	66.99 months (63.66 – 70.32)	1.70	<0.001
≥ 0.125	39.70 months (31.32 – 48.08)	4.28	
Overall	61.93 months (57.99 – 65.87)	2.01	

DFS, Disease-Free Survival; Disease free survival; MLNR, metastatic lymph node ratio.

## 4 Discussion

The assessment of prognosis in CC is crucial for determining treatment strategies and long-term disease management. In recent years, many researchers have proposed that the MLNR could be a prognostic factor in various malignancies, particularly gastrointestinal cancers (17–20). In colon cancer, the number of metastatic lymph nodes has also been shown to be a significant prognostic factor. In this study, MLNR emerged as a significant and independent predictor of recurrence and DFS. An MLNR threshold of 0.125 provided effective risk stratification, with higher ratios indicating substantially poorer outcomes. These findings support the integration of MLNR into postoperative risk assessment models. Our findings align with existing literature and offer new perspectives (21–24).

### 4.1 Prognostic significance of MLNR

MLNR emerges as a more sensitive prognostic indicator compared to the metastatic lymph node count in the TNM staging system. A meta-analysis has emphasized the prognostic value of MLNR in colon cancer, noting that patients with a higher MLNR tend to have worse survival outcomes (22). The current literature supports the use of lymph node ratio for colon cancer prognosis. In a systematic review and meta-analysis, increased lymph node ratio was associated with reduced OS (HR: 2.36) and DFS (HR: 3.71) (25). Similarly, another meta-analysis found significant associations between MLNR and both OS (HR: 1.91) and DFS (HR: 2.75) (24). Despite consistent findings across studies, there is no universally accepted cut-off value for MLNR. eta-analyses by Karjol et al. (2020) and Zhang et al. (2016) reported a wide range of thresholds for MLNR, varying from 0.05 to 0.6, with some studies categorizing patients into multiple risk groups. Frequently used single cut-off values include 0.1, 0.125, 0.2, and 0.3. Interestingly, the greatest consistency across studies has been observed when a threshold below 0.2 was applied (26, 27).

In our study, an MLNR cut-off of 0.125 was identified using ROC curve analysis and the Youden Index, providing an optimal balance between sensitivity and specificity for predicting recurrence. This threshold aligns with previously reported values and may offer more precise risk stratification within our cohort. Unlike studies that applied thresholds such as 0.1 or 0.2, our ROC-based cut-off of 0.125 offers a comparable prognostic power, though direct statistical comparisons were beyond the scope of our analysis. We found that patients with an MLNR≥0.125 had significantly higher odds of recurrence, with more than a 13-fold increase compared to those with lower MLNRs, as confirmed by adjusted multivariate analysis (OR: 13.07, 95% CI: 1.03- 166.57, p=0.048). Additionally, patients with lower MLNRs demonstrated significantly longer disease-free survival (p<0.001). These findings support the potential role of MLNR in recurrence risk assessment. Recent studies have reinforced the prognostic importance of MLNR in colon cancer. Ichhpuniani et al. and Harman Kamalı et al. demonstrated that a high MLNR is associated with reduced survival outcomes, supporting its integration into staging and treatment planning. Additionally, Wang et al. emphasized MLNR’s role in guiding personalized therapeutic decisions, while recent updates in the field have highlighted the relevance of MLNR-based risk stratification when considered alongside lifestyle-related factors (28–30). However, its clinical implementation requires external validation in larger, prospective cohorts. Until then, MLNR may serve as a useful adjunct in triaging patients for closer surveillance or adjuvant treatment planning.

### 4.2 MLNR and TNM staging

The TNM classification system considers the number of metastatic lymph nodes as one of the main factors determining prognosis. Guidelines recommend examining at least 12 lymph nodes in colon cancer surgery. However, this approach may overlook variations in the total number of lymph nodes examined, which can be influenced by surgeon and pathologist expertise, tumor location, and the urgency of surgery (31). Zhang and colleagues reviewed 33 studies analyzing different MLNR cut-off values and concluded that higher MLNR independently predicted survival in colon cancer patients, suggesting its inclusion in future staging systems (26). This finding is supported by numerous studies showing that MLNR is a better prognostic factor than the N stage (32–34). However, some studies have found that MLNR performs similarly to or less effectively than the N stage (35–37). Some researchers have proposed hybrid staging systems integrating MLNR with the TNM classification, which have shown superior outcomes (22, 38, 39). For example, Wang and colleagues demonstrated that patients classified as stage IIIB by TNM but with MLNR >30% had survival rates worse than those with MLNR ≤30%, more closely resembling stage IIIC patients. These findings suggest that MLNR may complement TNM staging in refining prognostic assessments, though further validation is warranted before recommending integration into standard staging algorithms.

### 4.3 Surgical and pathological factors

In our study, we found that emergency surgery and tumor localization significantly influenced mortality (p=0.023 and p=0.009, respectively). However, the type of surgery, histopathology, disease stage, and duration of hospitalization were not significantly associated with survival. Although the total lymph node count did not correlate with survival (p=0.273), MLNR was found to be a significant prognostic factor (p<0.001). This suggests that MLNR should be included as a complementary metric in the staging systems alongside TNM.

### 4.4 Adjuvant chemotherapy and MLNR

Adjuvant chemotherapy is a key component of colon cancer treatment and significantly impacts prognosis. The lymph node ratio (LNR) is an important determinant of both disease progression and adjuvant treatment planning. The relationship between MLNR and survival in colon cancer was first raised by Berger et al. (40). In another study examining 24,477 patients, MLNR was found to be a more accurate prognostic factor than the N stage. When the cut-off for MLNR was set at 0.2, patients with MLNR below this value had a survival rate of 81.1%, while those above it had a rate of 46.6%. Multivariate analysis showed that both thresholds were significant in predicting survival (41). Patients who received adjuvant therapy had a significantly lower risk of disease recurrence compared to those who did not (p=0.015). This indicates that adjuvant chemotherapy plays a critical role in colon cancer treatment and is effective in reducing the risk of recurrence. MLNR is a prognostic marker that can guide adjuvant therapy decisions. In multivariate analysis, patients with MLNR ≥ 0.125 had over 13-fold increased odds of recurrence (OR: 13.07, 95% CI: 1.03-166.57, p=0.048), whereas adjuvant therapy was associated with a 77% reduction in recurrence risk (OR: 0.23, 95% CI: 0.06-0.92, p=0.038). his finding suggests that adjuvant chemotherapy planning should be tailored more precisely for patients with elevated MLNR. Adjuvant chemotherapy decisions can be individualized by considering the MLNR cut-off value (0.125). Patients with MLNR <0.125 demonstrated better survival outcomes and lower recurrence rates. This suggests that MLNR can help identify the patient groups most likely to benefit from adjuvant chemotherapy. In multivariate analysis, adjuvant therapy and MLNR were identified as independent prognostic factors. The reduced recurrence risk among patients receiving adjuvant therapy (Exp(B): 0.234, p=0.038) underscores the efficacy of this treatment. Our results, supported by the literature, suggest that MLNR can guide adjuvant chemotherapy decisions, enabling more precise and individualized treatment planning.

### 4.5 Age and surgical factors

Age was found to be a significant factor for both mortality and recurrence in our study (p<0.001). Older patients are often burdened with more comorbidities and have limited surgical options, which may explain this association. However, in multivariate analysis, age was not an independent risk factor, suggesting potential collinearity with comorbidities and other factors. This observation is consistent with previous reports indicating that older patients with colorectal cancer tend to present with more advanced disease and experience poorer survival outcomes due to comorbidities and limited treatment options (42). The impact of emergency surgery on mortality has been previously reported in the literature. In a study by Hogan et al., it was noted that patients undergoing emergency surgery had worse prognosis, with limitations in adjuvant treatment planning (31). Recent evidence highlights that emergency colorectal resections are associated with significantly worse survival outcomes due to a combination of inadequate surgical conditions and elevated postoperative complication rates (43). Similarly, our study found that patients who underwent emergency surgery had lower survival rates (p=0.023).

### 4.6 Strengths and limitations of the study

This study has several limitations. Its retrospective, single-center design limits the generalizability of the findings and introduces potential selection bias. One of the limitations of our study is the lack of clinical data regarding lifestyle-related risk factors such as obesity, alcohol consumption, and smoking, which prevented their inclusion in the prognostic analysis. The moderate sample size may restrict subgroup analyses and reduce the precision of effect estimates, especially in rare subgroups. The absence of molecular and histopathological data, including microsatellite instability, KRAS/BRAF mutations, and tumor grade, limits the comprehensiveness of recurrence risk modeling and may contribute to residual confounding. A high rate of emergency surgeries (~40%) may have compromised the completeness of lymph node dissection. This could have influenced MLNR calculation, as urgent procedures often deviate from standard oncologic protocols. Additionally, including all lymph nodes regardless of their anatomical relevance might have overestimated total lymph node counts and underestimated MLNR. Future multicenter, prospective studies that incorporate detailed anatomical and tumor-specific lymph node categorization are necessary to validate MLNR as a reliable prognostic tool.

## 5 Conclusions

MLNR is a simple, accessible, and independent prognostic indicator in patients with surgically treated CC. In this study, MLNR ≥ 0.125 was associated with significantly higher recurrence risk and shorter DFS, even after adjustment for confounding variables. These findings suggest that MLNR can supplement TNM staging by providing additional risk stratification, particularly in cases where lymph node yield is limited or staging is borderline. MLNR may also help guide individualized decisions regarding adjuvant chemotherapy, especially in stage II and III patients, by identifying those at increased risk who may benefit from more intensive treatment or follow-up. Its integration into routine pathology reporting and multidisciplinary decision-making processes may improve prognostic accuracy and treatment planning in CC. Pending validation in larger studies, MLNR may be considered for future integration into pathology reporting and decision-making algorithms.

## Data Availability

The data analyzed in this study is subject to the following licenses/restrictions: The data that support the findings of this study are not openly available due to reasons of sensitivity and privacy are available from the corresponding author upon reasonable request. Data are located in controlled access data storage at Hitit University Faculty of Medicine Department of General Surgery. Requests to access these datasets should be directed to Ramazan Topcu, topcur58@gmail.com.
